# Parallel Mutations Result in a Wide Range of Cooperation and Community Consequences in a Two-Species Bacterial Consortium

**DOI:** 10.1371/journal.pone.0161837

**Published:** 2016-09-12

**Authors:** Sarah M. Douglas, Lon M. Chubiz, William R. Harcombe, F. Marty Ytreberg, Christopher J. Marx

**Affiliations:** 1 Department of Molecular and Cellular Biology, Harvard University, Cambridge, Massachusetts, United States of America; 2 Department of Organismic and Evolutionary Biology, Harvard University, Cambridge, Massachusetts, United States of America; 3 Faculty of Arts and Sciences Center for Systems Biology, Harvard University, Cambridge, Massachusetts, United States of America; 4 Department of Physics, University of Idaho, Moscow, Idaho, United States of America; 5 Institute for Bioinformatics and Evolutionary Studies, University of Idaho, Moscow, Idaho, United States of America; 6 Department of Biological Sciences, University of Idaho, Moscow, Idaho, United States of America; 7 Center for Modeling Complex Interactions, University of Idaho, Moscow, Idaho, United States of America; Georgia Institute of Technology, UNITED STATES

## Abstract

Multi-species microbial communities play a critical role in human health, industry, and waste remediation. Recently, the evolution of synthetic consortia in the laboratory has enabled adaptation to be addressed in the context of interacting species. Using an engineered bacterial consortium, we repeatedly evolved cooperative genotypes and examined both the predictability of evolution and the phenotypes that determine community dynamics. Eight *Salmonella enterica* serovar Typhimurium strains evolved methionine excretion sufficient to support growth of an *Escherichia coli* methionine auxotroph, from whom they required excreted growth substrates. Non-synonymous mutations in *metA*, encoding homoserine trans-succinylase (HTS), were detected in each evolved *S*. *enterica* methionine cooperator and were shown to be necessary for cooperative consortia growth. Molecular modeling was used to predict that most of the non-synonymous mutations slightly increase the binding affinity for HTS homodimer formation. Despite this genetic parallelism and trend of increasing protein binding stability, these *metA* alleles gave rise to a wide range of phenotypic diversity in terms of individual versus group benefit. The cooperators with the highest methionine excretion permitted nearly two-fold faster consortia growth and supported the highest fraction of *E*. *coli*, yet also had the slowest individual growth rates compared to less cooperative strains. Thus, although the genetic basis of adaptation was quite similar across independent origins of cooperative phenotypes, quantitative measurements of metabolite production were required to predict either the individual-level growth consequences or how these propagate to community-level behavior.

## Introduction

Multi-species microbial communities critical to human health [1,2], industrial production [3], and waste remediation [4,5] are governed by complex social dynamics. Predicting how these communities might respond to environmental changes requires an understanding of how species interactions change the evolution of new traits. Interspecies interactions, like competition or cooperation, complicate environmental selective pressures and decrease the predictive power of monoculture behavior [6,7]. For example, a cooperative species that excretes a beneficial cellular product, or “public good,” may alter the environment and thus alter the selective pressure on other species [8]. When this cooperation is costly to individual growth, but rewarded by cooperative behavior, a complex tension arises in which the relative costs and benefits and the structure of the interactions determine whether the cooperation is driven to fixation, coexists, or is eliminated. From the point of view of adaptation, it is unclear whether evolution in communities should be highly variable between instances, owing to the presence of multiple partners, or highly similar, because there are often few phenotypes that primarily govern the interaction [7,9,10].

Parallelism at various levels is common in experimental evolution. Replicate microbial populations under selection for growth in liquid medium have often evolved parallel phenotypes [7,8] or genotypes [11–14]. For within-species interactions, examples have suggested that genetic parallelism can be common, despite a resulting range in phenotypic consequences from these mutants [15,16]. Here, we address the spectrum of mutations that gave rise to metabolic cooperation between species, and how the resulting metabolite production phenotypes translated to individual costs or community dynamics.

**Fig 1:**
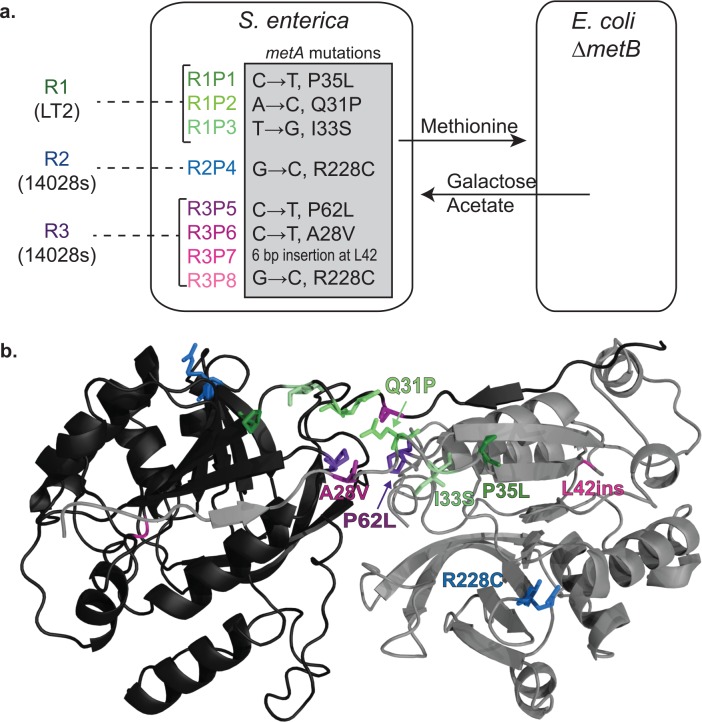
*S*. *enterica* cooperators evolved from ethionine resistant ancestors feature mutations in *metA*. a) *S*. *enterica* ethionine resistant strains (R strains) were co-cultured with *E*. *coli* methionine auxotrophs on lactose minimal media. Adaptive methionine excretion by evolved cooperators enabled growth of *E*. *coli ΔmetB*, which in turn excretes usable carbon for *S*. *enterica*. Non-synonymous substitutions in the *metA* gene, encoding homoserine trans-succinylase (HTS), are listed next to producer strain name. b) A homology model of residues 2–297 of *S*. *enterica* HTS was created using *Bacillus cereus* HTS. Each evolved cooperator features a mutation at one highlighted residue. The active site is shown with its cognate substrate homoserine.

Our model system is a synthetic community, or consortium, comprised of two-members: an *Escherichia coli* methionine auxotroph (Δ*metB*) and *Salmonella enterica* serovar Typhimurium [17]. Both members have a reciprocal requirement for the other to grow in lactose minimal media (Fig 1). *E*. *coli* excretes carbon byproducts that are consumed by *S*. *enterica*, as *S*. *enterica* cannot metabolize lactose. *S*. *enterica* must, in turn, evolve sufficient methionine excretion to support growth of the *E*. *coli* methionine auxotroph. By selecting in a spatially-structured environment, a cooperative methionine-producing *S*. *enterica* genotype was selected for, which could then sustain community growth either on petri dishes or in liquid media. In this initial instance of the evolution of cooperation methionine excretion by *S*. *enterica* was found to be individually costly, substantially decreasing the cooperator’s own growth rate compared to its ancestor [17].

Here we uncover the genetic basis of methionine excretion in the previously evolved cooperative *S*. *enterica*, as well as repeat the use of a spatially-structured environment to select for a series of new cooperative strains from different, closely-related *S*. *enterica* backgrounds. We first examined the underlying molecular changes in our *S*. *enterica* mutants and found that all evolved strains featured mutations in the *metA* gene, which encodes the first step of methionine biosynthesis, homoserine trans-succinylase (HTS) [18]. We used molecular modeling to predict how these mutations modified HTS folding and homodimer formation and found that most mutations increased the binding affinity for dimerization. We determined the effect of the *metA* mutations upon growth of consortia in well-mixed, liquid medium. While growth in liquid is distinct from the spatially structured environment in which the *S*. *enterica* evolved, it represents an informative phenotype and is more experimentally tractable. Although we found genetic parallelism, the various *metA* alleles gave rise to a wide range of methionine excretion levels. We found that methionine excretion correlated negatively with individual growth of *S*. *enterica*, but positively with the growth rate of the consortia and with the proportion of *E*. *coli* partner that the given strains could maintain. The extent a given allele commits the cell to cooperation thus simultaneously determines the individual-level growth costs, the community growth rate and the species ratio in the consortium.

## Results

### Replicate *S*. *enterica* cooperators were evolved from multiple starting genotypes

To explore the genetic and physiological range of adaptations underlying the rise of cooperation in our consortium, we repeatedly evolved methionine-producing *S*. *enterica* strains by selecting for their ability to support growth of their auxotrophic *E*. *coli* partner (Fig 1). Repeated attempts to evolve WT *S*. *enterica* to support community growth failed. We thus turned to a classic route to generating methionine overproduction in *S*. *enterica*, selection of resistance to ethionine, a toxic methionine analog [19,20]. Methionine production in *S*. *enterica* is tightly regulated at the level of transcription and translation via end-product inhibition [18,21,22]. Ethionine represses methionine biosynthesis via the same mechanism [21]. A previously characterized ethionine resistant (Eth^R^) strain [17] from *S*. *enterica* LT2 (Resistant strain, hereafter “R1”) did not excrete detectable levels of methionine, but was used as the ancestor for deriving an evolved cooperator that produced methionine (hereafter R1P1). We selected two new Eth^R^ backgrounds, “R2” and “R3,” from *S*. *enterica* 14028s. R1 is derived from the LT2 strain, while R2 and R3 were derived from 14028s; these two strains’ genome sequences differ by just 2% [23]. These three R strains were the ancestors for subsequent evolution of cooperation, and represent slightly distinct genetic backgrounds of the same serovar. None of these three strains excrete detectable amounts of methionine in the medium, but possess causative Eth^R^ mutations in methionine pathway transcriptional repressor *metJ* (Douglas *et al*., in preparation).

We evolved the new *S*. *enterica* cooperators by plating each R ancestor with *E*. *coli* Δ*metB* on lactose agarose plates, and screened for regions of lactose utilization by the *E*. *coli*. Once lactose utilization was observed, that consortium “colony” was streaked to retrieve isolates of each species, allowing cooperative *S*. *enterica* to be isolated from their region of origin on the lactose plate. By repeatedly replaying the evolution of methionine excretion from each R ancestor we obtained seven new, independent cooperators that could support community growth like the original strain (here “R1P1”) isolated from R1 previously (Fig 1).

### Genetic parallelism in cooperators evolved from different starting genotypes

In follow-up to preliminary genome sequencing of two of the *S*. *enterica* cooperators from different R ancestors (Fig 1; R1P1 and R2P4; sequence unpublished), targeted sequencing across the panel of evolved methionine-producing lineages revealed that they all contained mutations in *metA*, that encodes homoserine trans-succinylase (HTS) (Fig 1). All cooperators possessed either independent point mutations or an insertion in the coding region of HTS (Producer mutations; termed P1 to P8). Intriguingly, none appear within the active site (Fig 1). All but two substitutions fall within the first 62 amino acids of HTS. Each evolved *metA* allele is unique, with the exception of R228C in both *metA*^P4^ and *metA*^P8^ arising from an identical nucleotide substitution. Sequencing *metA* in the three ancestral R strains all showed the original *metA*^WT^ allele, indicating that each *metA* mutation immediately preceded the emergence of the cooperative phenotype.

### Molecular modeling suggests mutations tend to stabilize homodimer formation

**Fig 2:**
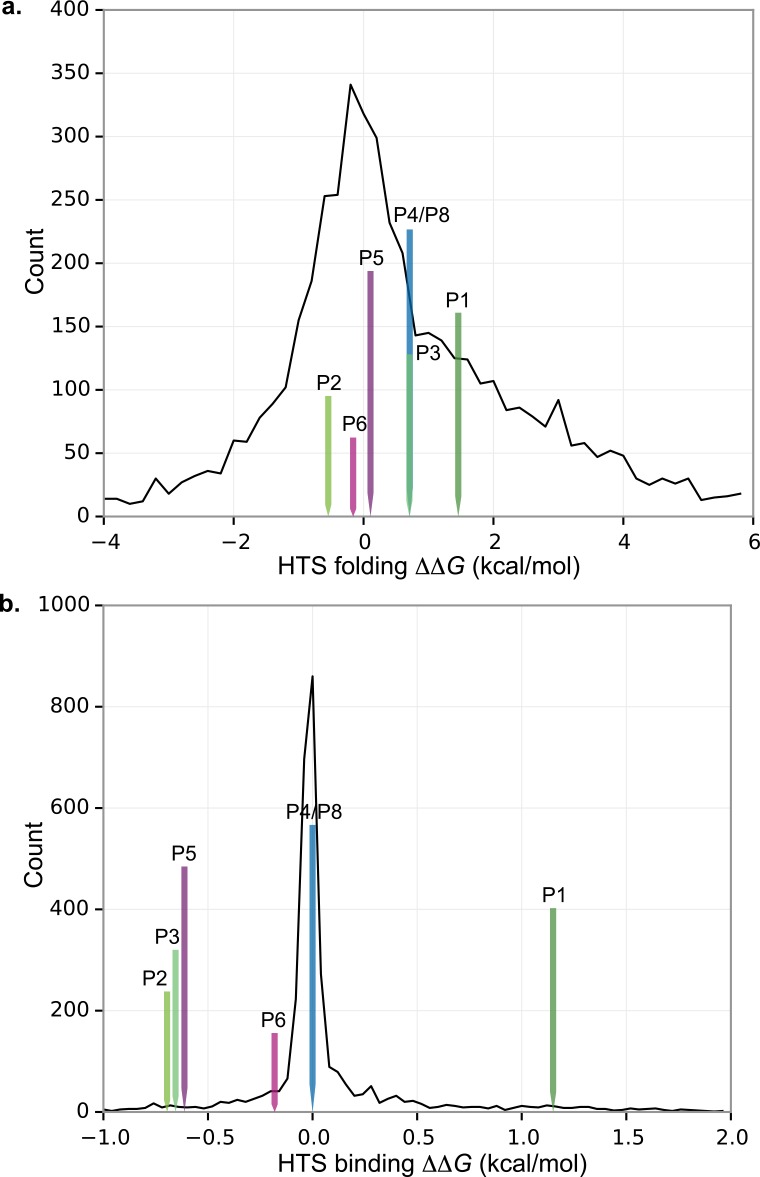
Molecular modeling suggests evolved HTS substitutions increase dimer stability. Stability changes were estimated using computer simulation. Histograms show stability changes *ΔΔG* for all possible amino acid changes for both folding and binding. Negative *ΔΔG* values correspond to stabilizing the folding of HTS or the dimer formation, and positive values destabilize folding or binding. The six single point mutations seen in this study are also indicated.

Molecular simulation suggests that the *metA* mutations are, at least in part, selected to stabilize homodimer formation of *metA*’s protein product, HTS, but not to stabilize folding of the monomer (Fig 2). Simulations were used to estimate the change in the protein folding and protein-protein binding stabilities for the six unique residue substitutions relative to wild type. To provide context for understanding the effects of these six mutations, all possible substitutions of HTS were calculated, that is, the changes in the stability were estimated for all 264 sites changing to the 19 other residue types (5016 mutations total). The figure shows histograms of the change in folding stability (Fig 2) and binding affinity (Fig 2) for all possible substitutions of HTS. The figure also indicates the six evolved substitutions seen in the experiments. The results show that two of the six evolved substitutions stabilize folding; this is consistent with chance since 38% of all possible mutations stabilize folding. The *p*-value for a Mann-Whitney U test of the effect of these six substitutions on folding stability was 0.38, consistent with the null hypothesis that they are random with respect to the distribution of all possible substitutions. By contrast, four evolved substitutions stabilize binding of the dimer, one (R228C) has no effect on stability since it is far from the binding interface, and one is destabilizing (P35L). The fraction of evolved substitutions that stabilize binding is much higher than one would expect by chance since only 29% of all possible mutations stabilize binding, and the corresponding *p*-value is 0.037.

### *metA* mutations are necessary for cooperative phenotype

**Fig 3:**
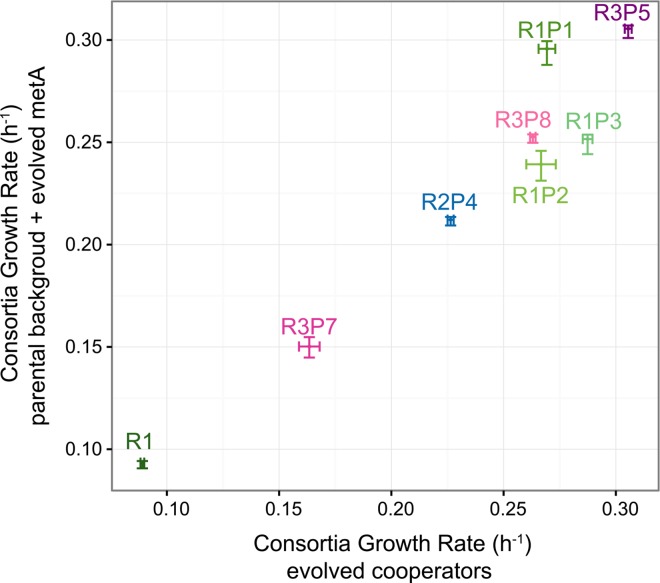
Substituting evolved *metA* alleles into R strains recapitulates cooperative phenotype. Consortia growth rates of R strains with evolved *metA* alleles correlates with growth rates of the original evolved strains (*p*-value < 0.001), including the control *metA*^WT^ in the R1 background demonstrating the sufficiency of *metA*^EVO^ alleles in the R backgrounds. Error bars indicate the standard error of three biological replicates.

To test the necessity of the *metA* mutations for cooperation, the ancestral *metA*^WT^ allele was substituted into each cooperative *S*. *enterica* mutant, and the ability to support growth in co-culture (consortia) with *E*. *coli* Δ*metB* was measured by growth in minimal liquid medium. Evolved *S*. *enterica* strains with the *metA*^WT^ allele reintroduced can no longer sustain growth in consortia with *E*. *coli* Δ*metB*. Conversely, the evolved *metA* alleles were not sufficient for consortia growth in the wild-type *S*. *enterica*, as they could not restore cooperation when placed alone into wild-type LT2 background (S1 Fig, Welch’s t test, *p* < 0.05 for both RP strains versus their P strain, and both P strains versus WT). Direct substitution of any of the evolved *metA* alleles into their ancestral R background, however, restored consortia growth rates to the levels seen from the original evolved producers (R^2^ = 0.930, Pearson correlation, *p* < 0.001, throughout using each biological replicate separately) (Fig 3). While the *metJ* mutations that provide ethionine resistance are essential to consortia growth (Douglas *et al*. in preparation) the phenotypic diversity arising from different *metA* mutations within the same Eth^R^ background indicated that *metA* mutations are the primary driver of cooperative disparity between *S*. *enterica* strains.

### Methionine excretion by evolved cooperators is highly variable

**Fig 4:**
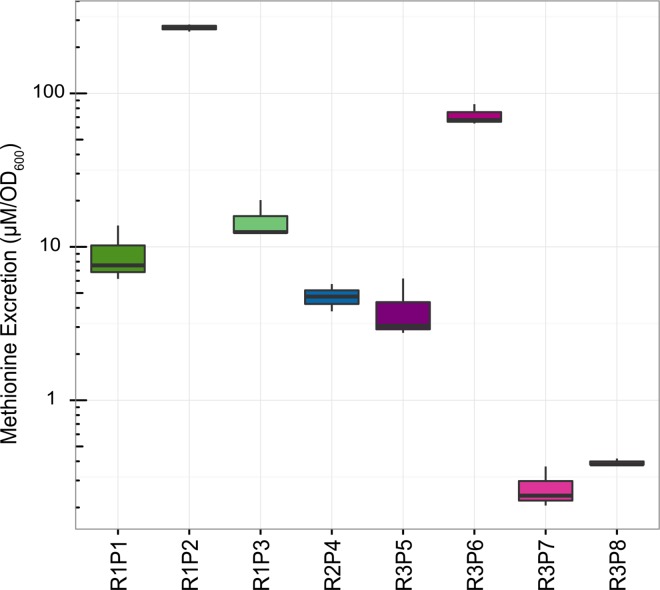
Methionine excretion by *S*. *enterica* producers varies 1000-fold. GC-MS quantification of methionine in conditioned media collected from evolved *S*. *enterica* strains at mid-log phase growth. Values are given as μM methionine per unit OD_600_. Each data point represents three biological replicates.

To quantify the variability of physiological consequences arising from parallel *metA* mutations in *S*. *enterica* evolved strains, methionine excretion was measured via gas chromatography/mass spectroscopy analysis of supernatant collected from mid-exponential phase *S*. *enterica* monocultures (Fig 4). Methionine excretion above the detection limit of 200 nM was not observed in conditioned media from the ancestral R strains. On the other hand, cooperators exhibited levels of production ranging from 0.272 to 268.2 μM in cultures normalized to an OD_600_ of 1.0 as a proxy for total biomass. This represents a 1000-fold range in methionine excretion despite similar genetic changes in the same locus.

### Methionine excretion correlates with group performance

**Fig 5:**
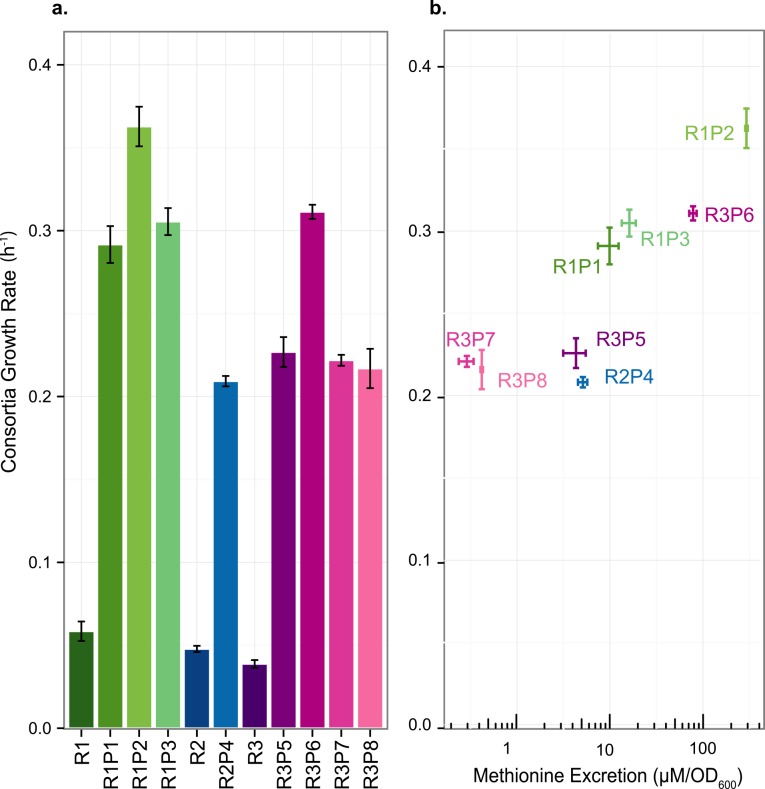
Increased consortia growth rate for evolved strains reflects increased *S*. *enterica* methionine excretion. a) Growth rate of cooperative *S*. *enterica* co-cultured with *E*. *coli* Δ*metB* in lactose minimal media. b) Growth rates of consortia containing evolved cooperators are positively correlated with methionine excretion by evolved *S*. *enterica* cooperators (*p*-value < 0.05). Error bars indicate standard error of three biological replicates.

In order to study the ecological consequences of the highly varied methionine excretion levels, consortia growth rates for ancestral and evolved *S*. *enterica* strains co-cultured with *E*. *coli* Δ*metB* were measured in lactose minimal media. Although the measurements are of the dynamics of the total OD_600_ of the consortia, it should be noted that these dynamics were exponential, and involved consistent ratios of species through growth once they reach an equilibrium during the first growth cycle (S2 Fig). Thus, although the proportion of species differs across consortia (see below), both partner species grow at the same rate within each consortium. Consortia containing R strains show slow but measurable growth (0.038–0.054 h^-1^), suggesting either basal *S*. *enterica* cell death or methionine leakage by the ancestral backgrounds is sufficient to maintain some *E*. *coli* growth. Consortia growth rates when using evolved strains, however, were much faster and varied from 0.209 to 0.362 h^-1^ (Fig 5, Welch’s t test, *p* < 0.05 for all comparisons of RP strains to the corresponding R strain). These growth rates were positively correlated (R^2^ = 0.773, Pearson correlation, *p* < 0.05) in a log-linear fashion with the cooperators’ methionine excretion rates previously measured (Fig 5).

### High methionine excretion is costly to individual growth

**Fig 6:**
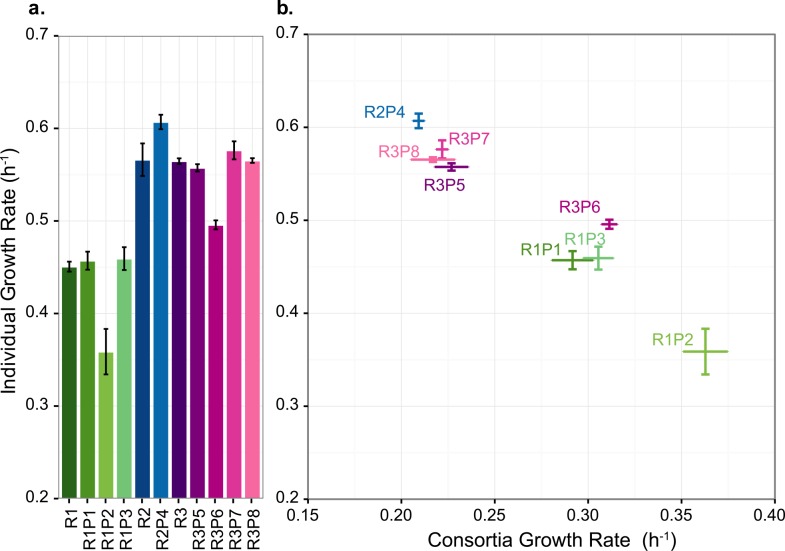
Decreases in individual growth of cooperators correlates with increases in consortia growth. a) Growth rates of *S*. *enterica* strains in galactose minimal media show decreased individual fitness in the most cooperative *S*. *enterica* strains. b) A negative correlation (*p*-value < 0.001) between individual and consortia growth rates of evolved cooperators demonstrates a trade-off between individual and cooperative selective pressures in these consortia.

To assess the extent to which methionine production generated trade-offs between individual growth capacity and community performance, as was observed for the first *S*. *enterica* cooperator [17], growth of all individual isolates and reconstituted consortia with *E*. *coli* Δ*metB* were characterized. *S*. *enterica* strain individual growth rates were measured in galactose minimal media (Fig 6). Galactose is one of the compounds excreted by the *E*. *coli* Δ*metB* partner while growing in lactose minimal medium supplemented with methionine [WRH and CJM, unpublished data]. Consistent with the effects within each lineage, the R1 strain displays the slowest initial growth, and moderately cooperative R1-derived strains grow similarly to R1, with the most cooperative (R1P2) growing more slowly. The R2 and R3-derived strains exhibited a relatively similar pattern, whereby the most cooperative strain grows most slowly. Examining all evolved cooperators collectively, however, it becomes clear that there is a fairly tight, negative linear correlation (R^2^ = 0.927, Pearson correlation, *p* < 0.001) between individual growth on galactose and consortia growth on lactose when methionine production is required (Fig 6). Furthermore, for the most cooperative strain, R1P2, consortia growth rate was indistinguishable from its individual growth rate (Welch’s t test, *p* = 0.894). The other cooperators all grew faster individually than when partnered in co-dependence with *E*. *coli*. Even when excluding R1P2 as an outlier of cooperation, correlation between cooperators’ individual and consortia growth rates is maintained (R^2^ = 0.874, Pearson correlation, *p* < 0.05).

### The most cooperative *S*. *enterica* strains support highest ratio of *E*. *coli* partner

**Fig 7:**
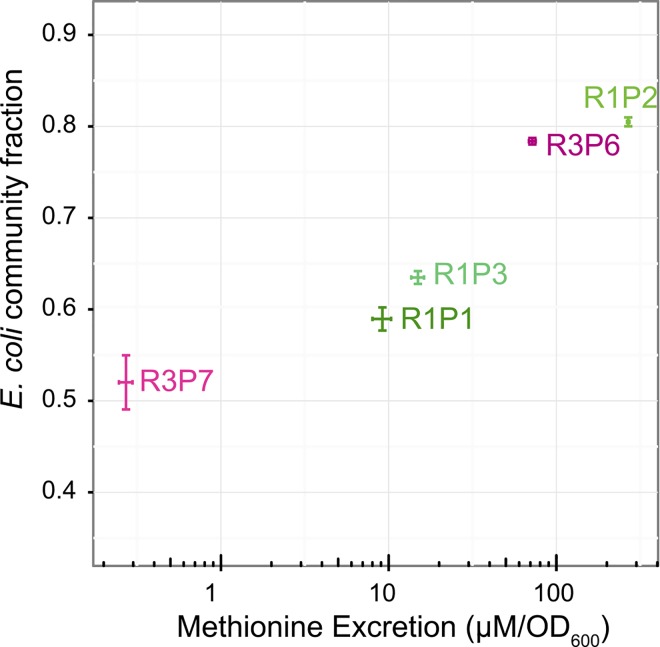
Consortia composition in liquid communities correlates with *S*. *enterica* methionine excretion. YFP-labeled *S*.*enterica* and CFP-labeled *E*. *coli* Δ*metB* grown in liquid were sampled at completion of growth, and consortia composition determined via flow-cytometry. *E*. *coli* composition of each community correlates with methionine excretion of *S*. *enterica* within that community (*p*-value < 0.05). Error bars represent standard error of three biological replicates.

Given the wide differences in methionine excretion, individual growth, and community performance, we further examined the consequence of each evolved *metA* allele upon the species ratio in our consortia. A subset of the *S*. *enterica* cooperators were fluorescently-labeled with yellow fluorescent protein, YFP, and co-cultured with cyan fluorescent protein, or CFP-labeled *E*. *coli* Δ*metB* in both liquid and solid media. *S*. *enterica* methionine excretion correlates with *E*. *coli* percentages in liquid culture grown to stationary phase (R^2^ = 0.937, Pearson correlation, p < 0.05) (Fig 7), showing that individual behavior can predict this ecological trait.

## Discussion

By selecting for cooperative phenotypes multiple times with a synthetic, two-species consortium, we determined the degree of both the genetic and phenotypic parallelism for mutations that allowed between-species cooperation to emerge. Within the context of a multi-species, spatially-structured community, the environmental interactions are presumed to be more complex than those faced by individual, planktonic cells in well-mixed media. It might be expected that the range of adaptive mutations similarly increase just as biotic interactions often increase diversification. Because the initiation of cooperation was immediately observable on petri dishes due to the co-metabolic conversion of X-gal caused by lactose growth, our experimental design allowed us to obtain first step beneficial mutations as they occurred, before they competed with other possible mutations for fixation. By avoiding clonal interference [24] with other consortia-inducing mutations that generated separate colonies, it seems more likely that the genetic parallelism we observed was due to what mutations can occur, not just those whose selective coefficients are sufficiently large to outcompete others occurring in a given population size [25,26]. We also avoid the possible confounding effects of long-term positive interactions between genotypes such as cross-feeding that can lead to clonal reinforcement [27,28].

We found that the genetic basis for *S*. *enterica* to initiate a novel cooperative interaction was quite narrow, similar to previous work on within-species cooperation [29]. Why might natural selection have acted so narrowly? In this case, a relatively dramatic change in phenotype was required for consortia growth, and the ability of consortia to grow was limited by a single factor: methionine production. Given that regulation of methionine production (like many amino acid biosynthetic pathways) is strongly controlled at the first step of the five enzymes involved, HTS encoded by *metA* exerts a high degree of metabolic control [30] over this phenotype under these conditions. This suggests that when a single metabolic compound is the currency of exchange, changes at one critical point in central metabolism of one organism might be sufficient for large-scale ecological changes.

An additional factor that may have contributed to the observed genetic parallelism was that the evolved phenotype was likely a loss of function of an active negative regulation mechanism upon HTS levels. Previous work has demonstrated that the N-terminus region of the *E*. *coli* HTS destabilizes the protein via energy-dependent proteolysis [31]. Deletion or replacement of the first 68 amino acids of HTS in *E*. *coli* dramatically increased HTS half-life. Several of our P mutations were in HTS in *S*. *enterica;* the homologs in the two species share 95% amino acid identity. This suggests that the mutations we observed in *S*. *enterica* acted to increase protein stability. Consistent with this hypothesis, two independent mutations that stabilized the *E*. *coli* HTS and increased heat tolerance, S61T and I229T [32], neighbor amino acid substitutions in *metA*^P4^, *metA*^P5^, and *metA*^P8^. The R228C substitutions in *metA*^P4^ and *metA*^P8^ occur in the C-terminus region where other mutations have been found to desensitize HTS to allosteric inhibition of methionine [33]. Collectively, these prior experiments are consistent with the idea that any of a potentially wide range of mutations that increase HTS stability or reduce its sensitivity to end-product inhibition could lead to cooperation, and thus parallelism at the level of the gene.

While the probability and/or the magnitude of beneficial mutations in *metA* led to genetic parallelism, the resulting alleles conferred a wide spectrum of phenotypes. Genetic parallelism has been a fairly common finding from evolution of microbes in the laboratory, as well as environments such as chronic infections [34], but there are relatively few examples where consistency of phenotype between alleles has been tested [12,35–37]. Similar to work for within-species cooperation enabled by mutations affecting siderophore production [29] or a transcriptional regulator [15], we found that parallel mutations in *metA* resulted in variation in methionine production over three orders of magnitude. Furthermore, methionine production explained most of the variation in decreased growth rate on galactose caused by these alleles.

Though the link between parallel genetic changes and the resulting diversity in methionine excretion is unclear, this is one of few examples where the phenotypes of an individual microbial species have been shown to quantitatively correlate with community phenotypes in a complex assemblage [7,9,10,38]. The correlations between production, individual growth and community performance suggest that, at least in the absence of refinement through additional mutations, metabolite production itself governs the benefits and costs of cooperation: the more methionine you make, the slower you grow alone. It is also apparent, however, that methionine production was only substantially costly at high levels. The four isolates producing below 5 μM/OD_600_ grew alone with less than a 10% defect, whereas the four isolates producing 8 μM/OD_600_ had growth defects of 20–40%. A second general trend we found was that the more cooperative a strain is, the lower its observed equilibrium frequency in the community. This result parallels findings by Momeni et al 2013 in a single species [39]. In our work the most cooperative *S*. *enterica* strain (R1P2) is illustrative. Its consortia grows at a rate indistinguishable from its individual maximal growth rate, suggesting that it is not limited by growth substrate coming from *E*. *coli*, but that the *E*. *coli* is solely dependent upon a small, slow population of R1P2 for support. For all other *S*. *enterica* cooperators, they are capable of faster growth than their corresponding consortia with *E*. *coli*, suggesting that they are limited by growth substrate from *E*. *coli*. Since the same *E*. *coli* partner strain was present in all consortia, this in turn suggests that *E*. *coli* was limited in their own growth by the insufficient provisioning of methionine from their respective *S*. *enterica* partner strains. Simply by measuring the methionine output by the *S*. *enterica* partner, predictions about higher-level community characteristics within this community can be made with some accuracy.

The ability to correlate community composition with future behavior grows increasingly important as we come to rely on genetic sequencing of complex, multi-species microbial systems as a first pass estimator of community characteristics. Accurate inference of ecological behavior becomes more difficult as interactions between shifting selective pressures, genetic changes, and phenotypic expression within an organism are further complicated by species interactions. Metagenomic investigation of known interactions such as adaptive diversification have suggested that even time-series sequence data offer surprisingly few obvious clues of the underlying ecology [27,40]. We have shown here that even highly repeatable genetic adaptations in a dynamic community may translate into an unexpected array of phenotypes; our sequence data alone did not reflect the range of observed methionine excretion. Yet consistent evidence of physiological trade-offs and repeatable community dynamics gives some credence to the idea that the trajectory of even a species-rich evolving community might become predictable once the relevant phenotypes are characterized.

## Materials and Methods

### Growth media and strains

The experimental system consisted of an *E*. *coli* methionine auxotroph (*E*. *coli* Δ*metB*) and an ethionine resistant *S*. *enterica* partner. An *E*. *coli* strain K12 BW25113 with a *metB* knockout was obtained from the Keio collection, with lactose metabolism restored as described [17]. Ethionine resistant *S*. *enterica* mutants from LT2 and 14028s backgrounds were selected as described [17]. Cultures were grown in 1X (liquid media) or 0.5X (solid media) “Hypho” minimal media containing C7 trace metal mix [41] and were supplemented with either 0.1% (liquid media) or 0.05% (solid media) galactose or lactose. Antibiotic concentrations used were: 50 μg/mL ampicillin, 25 μg/mL chloramphenicol. All antibiotics and chemicals were obtained from Sigma Aldrich (St. Louis, MO) unless otherwise noted. All strains are listed in S1 Table.

### Evolution of methionine excreting *S*. *enterica* mutants

The two-step selection process for evolving a methionine-excreting *S*. *enterica* LT2 mutant is described in Harcombe 2010. Initial selection on ethionine, a competitive methionine analog, was again utilized to create new evolutionary ancestors from *S*. *enterica* serovar Typhimurium 14028s. Co-culturing of ethionine-resistant *S*. *enterica* and *E*. *coli* Δ*metB* on lactose Hypho agarose plates to select for methionine excretion proceeded as described [17]. Briefly, 10^7^ ethionine-resistant *S*. *enterica* cells grown overnight in Hypho galactose liquid media from a single colony were plated on lactose Hypho agarose plates with 10^7^ *E*. *coli* Δ*metB* cells grown overnight in Hypho lactose liquid media supplemented with 100 μM methionine. The bacteria were allowed to grow for 3 days at 37°C before the cells were scraped off, vortexed, and added to a fresh plate at 1/10 dilution. This second plate was allowed to grow for 3–5 days, or until colonies indicating co-growth of *S*. *enterica* and *E*. *coli* appeared. *S*. *enterica* strains were isolated from these colonies and tested for co-growth with *E*. *coli* Δ*metB* in Hypho lactose liquid and solid media.

### Genomic sequencing

Genomic DNA from *S*. *enterica* strains LT2, 14028s, and R2P4 was extracted from lysed cells using Wizard Genomic DNA Purification Kit (Promega, Madison, WI), and prepared for Illumina sequencing using the TrueSeq kit (Illumina, San Diego, CA). Samples were sent to The Microarray and Genomic Analysis Core facility at the University of Utah for sequencing on Illumina HiSeq 2000 sequencer, and aligned and analyzed using breseq, with all default users settings other than enabled polymorphism prediction [42] (http://www.barricklab.org/breseq).

### Plasmids

Gene replacement amplicons utilized the chloramphenicol-containing pKD32 plasmid as a template, and pKD46 as a helper plasmid [43].

### Structural analysis of HTS

Homology modeling of HTS (residues 2–297) from *S*. *enterica* was performed in SWISS-MODEL [44,45] using homoserine o-succinyltransferase from *Bacillus cereus* (PDB 2ghrA; 49% amino acid identity) as the template. Alignment of model to 2h2wA, as well as model visualization were performed in PyMOL [46].

### Molecular modeling of *metA* mutations

Changes in protein folding stabilities and protein-protein binding stabilities were estimated using FoldX [47,48]. FoldX was chosen for this study to balance accuracy and speed [49,50]. Given the large number of substitutions studied here, it is not possible to use accurate statistical mechanical approaches such as all atom molecular dynamics simulation as done in previous studies [51,52]. Using an in-house script a total of 5016 mutations were generated and analyzed (each of the 264 residues in the protein structure mutated to one of other 19 types) for both the unbound monomer and bound dimer forms. The monomer and dimer structures were initially equilibrated six times in succession using "RepairPDB" to obtain a minimalized conformation, and mutant forms of both monomer and dimer were generated using “BuildModel”. Protein folding stabilities were then estimated using "Stability" on the monomer structures and protein-protein binding stabilities were estimated using “AnalyseComplex” on the dimer structures.

The *p*-values were calculated based on the null hypothesis that the folding and binding stabilities of the six experimental substitutions are random samples from the distribution of all possible substitutions. A non-parametric sum of ranks method (Mann-Whitney U test) was used for both folding and binding. The 5016 stability values for stability were first ranked in order of most stabilizing (smallest ΔΔG value) to least stabilizing (largest ΔΔG value). Tied values received a rank equal to the average of the ranks they span. One million random samples of six ΔΔG values were then drawn from the distribution. The sum of ranks were calculated for each draw and compared to the sum of ranks for the six experimental substitutions. The *p*-value was calculated as the number of draws where the sum of ranks for the random draw was less than or equal to the sum of ranks for the experimental substitutions. The calculation was performed five times for both binding and folding to ensure consistent results.

### Gene Disruption

Gene disruptions were performed using the method of Datsenko and Wanner [43] with modifications described by Ellermeier *et al*. [53]. A selectable chloramphenicol marker (*cat*) flanked by 40 bp of the region surrounding the coding region of *metA* was constructed via PCR using plasmid pKD32 as template [38] and primers listed in S2 Table. PCR products were cleaned using QiaQuick PCR Purification kit (Qiagen) and electroporated into electrocompetent *S*. *enterica* cells carrying lambda Red helper plasmid pKD46 [43]. Cells were suspended in LB and recovered for 1 hr shaking at 37°C before being spread on selective media. Cells were purified once more selectively at 37°C before Δ*metA*::*cat* insertion was verified via PCR.

### P22 Transduction

To create lysates for P22 transduction, *S*. *enterica* donor strains were grown overnight, and then diluted 1:500 in 5mL LB+cat with 150 μL P22 HT *int* lysate stock and grown with shaking at 37°C for approximately 6 hours. After vortexing with 1 mL chloroform to kill remaining donor cells and centrifuging 10 minutes at 4550 x *g* to remove debris, lysate was stored at 4°C for up to 3 years. 200 μL overnight culture of recipient *S*. *enterica* strains were incubated with 100 μL lysate for 25 minutes at room temperature, rinsed twice with 100 mM sodium citrate LB, plated onto selective media, and grown overnight. After purifying once more selectively at 37°C, strains were cross-streaked against lytic P22 H5 lysate to test for remaining presence of phage.

### Allele Replacement

Native *metA* loci were deleted via P22 transduction of Δ*metA*::*cat* and selection on LB+ chloramphenicol. Cured Δ*metA*::*cat* strains received replacement loci via P22 transduction of donor strains containing the desired new *metA* allele and selection on glucose minimal media. Amplification and sequencing of new *metA* locus confirmed no additional mutations were introduced.

### Methionine measurements

Methionine measurements via GC-MS closely followed the method of Zamboni *et al*. [54]. To obtain conditioned media samples, overnight cultures of *S*. *enterica* strains were transferred at a dilution ranging from 50- to 200-fold into 30 mL galactose minimal media, and grown to mid-log phase in shaking flasks at 30°C. The *E*. *coli* release galactose and acetate, however, we chose to solely provide galactose to prevent the complexity of diauxic growth in batch culture. Galactose concentrations were also chosen to provide for sufficient growth of the *S*. *enterica* strains, rather than to imitate the levels experienced during co-culture experiments, and were kept the same across these strains. It should also be noted that we are using OD_600_ as a proxy for total biomass, rather than cell number per se, as it is the former that we wish to normalize production to. Samples were collected at mid-log phase to prevent depletion of exogenous methionine by *S*. *enterica* catabolism in stationary phase. Cultures were then pelleted for 10 minutes at 4°C and 4550 x *g*, and then filtered to complete removal of cells. After freezing at -80°C, thawed conditioned media was passed through solid phase extraction Chromaband Easy columns per manufacturer directions (Macherey-Nagel) and eluted in methanol. After removal of methanol in a vacuum centrifuge, and resuspension in 40 μL dimethylformamide, samples were placed into glass vials and derivatized for 1 hour at 85°C with 40 μL *N*-(tertbutyldimethylsilyl)-*N*-methyltrifluoroacetamide with 1% (wt/wt) tertbutyldimethyl-chlorosilane (Sigma). Derivatized samples were then immediately injected into a Shimadzu QP2010 GCMS (Columbia, MD). The injection source was 230°C. The oven was held at 160°C for 1 minute, increased to 310°C by 20°C min^-1^, and held at 310°C for 30 seconds. Column flow rate was 1.04 mL/min and the split ratio was 1.0. The column was a 30 m Rxi-1ms (Restek, Bellefonte, PA). Results were analyzed in GC-MS Postrun Analysis (Verison 2.70, Shimadzu, Kyoto, Japan). Methionine peak area was compared to an internal standard of 100 μM isoleucine added directly to the supernatants after thawing (S3 Fig). Experimental methionine concentrations were determined by a calibration curve, and then divided by the conditioned media culture’s final optical density as a proxy for total biomass. Methionine concentrations are given as μM/OD_600_ and represent a minimum of three biological replicates, each with three technical replicates.

### Growth rate analysis

Strains were acclimated by inoculating single colonies into 640 μL medium in Costar 48-well cell culture plates (Product #3548, Corning Life Sciences, Tewksbury, MA) and placed in a humidified plate shaking tower (Liconic) at 30°C overnight. For growth rate measurements, strains were then transferred with a 1:1280 dilution into fresh medium and returned to the shaking tower. Optical densities were obtained every 30 minutes (individual growth) or 60 minutes (consortia growth) on a Wallac Victor 2 plate reader (Perkin-Elmer) until cultures reached saturation, using an automated measurement system [55]. Growth rates were quantified by fitting the data to an exponential growth model using custom analysis software [41] and averaging a minimum of three biological replicates.

### Consortia Composition

*S*. *enterica* strains were labeled with a single chromosomal copy of Venus via transduction from LC1589 *E*. *coli* strain was labeled with a single chromosomal copy of Cerulean via P1 transduction from strain LC1511. Growth rates of the resulting fluorescent strains did not differ significantly from unlabeled strains. Hypho lactose liquid cultures were inoculated as described above, and 450 μL samples from replicate cultures in the same 48-well plate were collected at inoculation, mid-log, and stationary phase, and frozen at -80°C in 10% DMSO. 25 mL Hypho lactose agarose plates were inoculated with 50 μL of each YFP *S*. *enterica* strain and 50 μL of the CFP *E*. *coli* strain and incubated at 30°C for 72 hours before transfer. To transfer, 1 ml of Hypho added to the surface of plates, scraped, vortexed, and then 100 μL was transferred to new plates. Remaining culture from initial inoculation and each subsequent scraping were frozen at -80°C in 10% DMSO until analysis. Cytometry samples were diluted 1:1000 in PBS and analyzed on BD LSRFortessa II (BD, San Jose, CA). CFP-labeled *E*. *coli* and YFP-labeled *S*. *enterica* monoculture and non-fluorescent *S*. *enterica* were used determine cutoffs for 40,000 events gathered using the 405 nm and 488 nm lasers.

## Supporting Information

S1 Fig Consortia growth dependence upon *metA* alleles of *S*. *enterica*. Wild-type with the evolved alleles of *metA* cooperate more than with the original allele, but significantly less than the respective evolved isolates. Asterisks indicate presence of native allele of that background (i.e., no substitution).
(DOCX)

S2 Fig Consortia composition reaches an equilibrium with time. YFP-labeled *S*. *enterica* and CFP-*labeled E*. *coli* Δ*metB* grown liquid media were sampled at various time points, and consortia composition determined via flow-cytometery. Each data point represents three biological replicates, each with three technical replicates.
(DOCX)

S3 Fig Example GC-chromatogram of derivatized amino acids from cooperator spent media. This sample is one biological replicate of R1P2, with the methionine and isoleucine peaks clearly visible. Fragment patterns at each peak match those of identified derivatized amino acids. Area under each peak corresponds to amino acid quantity.
(DOCX)

S1 Table Strain and plasmid list. (DOCX)

S2 Table Primers used in gene disruptions, replacements, and epitope tagging. (DOCX)

## References

[1] The Human Microbiome Consortium (2012) Structure, function and diversity of the healthy human microbiome. Nature 486: 207–214. Available: http://www.pubmedcentral.nih.gov/articlerender.fcgi?artid=3564958&tool=pmcentrez&rendertype=abstract. Accessed 19 February 2014. 10.1038/nature11234 22699609 PMC3564958

[2] Ma B, Forney LJ, Ravel J (2012) The vaginal microbiome: rethinking health and diseases. Annu Rev Microbiol 66: 371–389. 10.1146/annurev-micro-092611-150157 The. 22746335 PMC3780402

[3] Smid EJ, Lacroix C (2013) Microbe-microbe interactions in mixed culture food fermentations. Curr Opin Biotechnol 24: 148–154. Available: http://www.ncbi.nlm.nih.gov/pubmed/23228389. Accessed 11 March 2014. 10.1016/j.copbio.2012.11.007 23228389

[4] Wagner M, Loy A, Nogueira R, Purkhold U, Lee N, Daims H. (2002) Microbial community composition and function in wastewater treatment plants. Antonie Van Leeuwenhoek 81: 665–680. Available: http://www.ncbi.nlm.nih.gov/pubmed/12448762. 12448762 10.1023/a:1020586312170

[5] Paliwal V, Puranik S, Purohit HJ (2011) Integrated perspective for effective bioremediation. Appl Biochem Biotechnol 166: 903–924. Available: http://link.springer.com/10.1007/s12010-011-9479-5. Accessed 7 February 2014. 10.1007/s12010-011-9479-5 22198863

[6] Liow LH, Van Valen L, Stenseth NC (2011) Red Queen: from populations to taxa and communities. Trends Ecol Evol 26: 349–358. Available: http://www.ncbi.nlm.nih.gov/pubmed/21511358. Accessed 20 March 2014. 10.1016/j.tree.2011.03.016 21511358

[7] Lawrence D, Fiegna F, Behrends V, Bundy JG, Phillimore AB, Bell T, et al (2012) Species interactions alter evolutionary responses to a novel environment. PLoS Biol 10: e1001330 Available: http://www.pubmedcentral.nih.gov/articlerender.fcgi?artid=3352820&tool=pmcentrez&rendertype=abstract. Accessed 24 March 2014. 10.1371/journal.pbio.1001330 22615541 PMC3352820

[8] West S, Griffin AS, Gardner A, Diggle SP (2006) Social evolution theory for microorganisms. Nat Rev Microbiol 4: 597–607. Available: http://www.ncbi.nlm.nih.gov/pubmed/16845430. Accessed 20 February 2014. 16845430 10.1038/nrmicro1461

[9] Hillesland KL, Stahl DA (2010) Rapid evolution of stability and productivity at the origin of a microbial mutualism. Proc Natl Acad Sci USA. 107(5): 2124–2129. 10.1073/pnas.0908456107 20133857 PMC2836651

[10] Andrade-Dominguez A, Salazar E, Vargas-Lagunas MC, Kolter R, Encarnacio S. (2014) Eco-evolutionary feedbacks drive species interactions. ISME. 8: 1041–1054. 10.1038/ismej.2013.208 PMC3996687 24304674

[11] Rainey PB, Travisano M (1998) Adaptive radiation in a heterogeneous environment. Nature 32: 69–72. 10.1038/27900 9665128

[12] Chou H-H, Marx CJ (2012) Optimization of gene expression through divergent mutational paths. Cell Rep 1: 133–140. Available: http://www.pubmedcentral.nih.gov/articlerender.fcgi?artid=3407975&tool=pmcentrez&rendertype=abstract. Accessed 25 March 2014. 10.1016/j.celrep.2011.12.003 22832162 PMC3407975

[13] Woods R, Schneider D, Winkworth CL, Riley MA, Lenski RE (2006) Tests of parallel molecular evolution in a long-term experiment with *Escherichia coli*. Proc Natl Acad Sci U S A 103: 9107–9112. Available: http://www.pubmedcentral.nih.gov/articlerender.fcgi?artid=1482574&tool=pmcentrez&rendertype=abstract. 16751270 10.1073/pnas.0602917103 PMC1482574

[14] Tenaillon O, Rodríguez-Verdugo A, Gaut RL, McDonald P, Bennett AF, Long AD, et al (2012) The molecular diversity of adaptive convergence. Science 335: 457–461. Available: http://www.ncbi.nlm.nih.gov/pubmed/22282810. Accessed 19 March 2014. 10.1126/science.1212986 22282810

[15] Kim W, Racimo F, Schluter J, Levy SB, Foster KR (2014) Importance of positioning for microbial evolution. Proc Natl Acad Sci U S A 111: E1639–E1647. Available: http://www.ncbi.nlm.nih.gov/pubmed/24715732. 10.1073/pnas.1323632111 24715732 PMC4000849

[16] MacLean RC, Buckling A (2009) The distribution of fitness effects of beneficial mutations in *Pseudomonas aeruginosa*. PLoS Genet 5: e1000406 Available: http://dx.plos.org/10.1371/journal.pgen.1000406. 10.1371/journal.pgen.1000406 19266075 PMC2646133

[17] Harcombe W (2010) Novel cooperation experimentally evolved between species. Evolution 64: 2166–2172. Available: http://www.ncbi.nlm.nih.gov/pubmed/20100214. Accessed 3 March 2014. 10.1111/j.1558-5646.2010.00959.x 20100214

[18] Old IG, Phillips SE, Stockley PG, Saint Girons I (1991) Regulation of methionine biosynthesis in the Enterobacteriaceae. Prog Biophys Mol Biol 56: 145–185. Available: http://www.ncbi.nlm.nih.gov/pubmed/1771231. 1771231 10.1016/0079-6107(91)90012-h

[19] Chubiz L, Douglas S, Harcombe W (2014) Combining engineering and evolution to create novel metabolic mutualisms between species Engingeering and Analzying Multicellular Systems. New York: Springer pp. 39–49. 10.1007/978-1-4939-0554-6_3 24838877

[20] Lawrence DA, Smith DA (1968) Regulation of methionine synthesis in *Salmonella typhimurium*: Mutants resistant to inhibition by analogues of methionine. 58: 473–492. 4879382 10.1093/genetics/58.4.473 PMC1224492

[21] Lawrence DA (1972) Regulation of methionine feedback-sensitive enzyme in mutants of *Salmonella typhimurium*. J Bacteriol 109: 8–11. Available: http://www.pubmedcentral.nih.gov/articlerender.fcgi?artid=247244&tool=pmcentrez&rendertype=abstract. 4550678 10.1128/jb.109.1.8-11.1972 PMC247244

[22] Hobson AC, Smith DA (1973) *S*-adenosylmethionine synthetase in methionine regulatory mutants of *Salmonella typhimurium*. Mol Gen Genet 126: 7–18. Available: http://www.ncbi.nlm.nih.gov/pubmed/4591373. 4591373 10.1007/BF00333477

[23] Jarvik T, Smillie C, Groisman EA, Ochman H (2010) Short-term signatures of evolutionary change in the *Salmonella enterica* serovar typhimurium 14028 genome. J Bacteriol 192: 560–567. Available: http://www.pubmedcentral.nih.gov/articlerender.fcgi?artid=2805332&tool=pmcentrez&rendertype=abstract. Accessed 26 March 2014. 10.1128/JB.01233-09 19897643 PMC2805332

[24] Gerrish PJ, Lenski RE (1998) The fate of competing beneficial mutations in an asexual population. Genetica 102–103: 127–144. Available: http://www.ncbi.nlm.nih.gov/pubmed/9720276. 9720276

[25] Rozen DE, de Visser JA, Gerrish PJ (2002) Fitness effects of fixed beneficial mutations in microbial populations. Curr Biol 12: 1040–1045. 12123580 10.1016/s0960-9822(02)00896-5

[26] Hegreness M, Shoresh N, Hartl D, Kishony R (2006) An equivalence principle for the incorporation of favorable mutations in asexual populations. Science 311: 1615–1617. 10.1126/science.1122469 16543462

[27] Herron MD, Doebeli M (2013) Parallel evolutionary dynamics of adaptive diversification in *Escherichia coli*. PLoS Biol 11: e1001490 Available: http://www.pubmedcentral.nih.gov/articlerender.fcgi?artid=3576414&tool=pmcentrez&rendertype=abstract. Accessed 28 May 2014. 10.1371/journal.pbio.1001490 23431270 PMC3576414

[28] Kinnersley M, Wenger J, Kroll E, Adams J, Sherlock G, Rosenzweig F. (2014) *Ex uno plures*: clonal reinforcement drives evolution of a simple microbial community. PLoS Genet 10: e1004430 Available: 10.1371/journal.pgen.1004430 24968217 PMC4072538

[29] Kümmerli R, Santorelli L a., Granato ET, Dumas Z, Dobay A, Griffin AS, et al (2015) Co-evolutionary dynamics between public good producers and cheats in the bacterium *Pseudomonas aeruginosa*. J Evol Biol. Available: http://doi.wiley.com/10.1111/jeb.12751. 10.1111/jeb.12751 26348785

[30] Kascer H, Burns JA (1973) The control of flux. Symp Soc Exp Biol 27:65–104. 4148886

[31] Biran D, Gur E, Gollan L, Ron EZ (2000) Control of methionine biosynthesis in *Escherichia coli* by proteolysis. Mol Microbiol 37: 1436–1443. 10998174 10.1046/j.1365-2958.2000.02097.x

[32] Mordukhova EA, Lee H-S, Pan J-G (2008) Improved thermostability and acetic acid tolerance of *Escherichia coli* via directed evolution of homoserine *O*-succinyltransferase. Appl Environ Microbiol 74: 7660–7668. Available: http://www.pubmedcentral.nih.gov/articlerender.fcgi?artid=2607180&tool=pmcentrez&rendertype=abstract. Accessed 3 March 2014. 10.1128/AEM.00654-08 18978085 PMC2607180

[33] Usuda Y, Kurahashi O (2005) Effects of deregulation of methionine biosynthesis on methionine excretion in *Escherichia coli*. Appl Environ Microbiol 71: 3228–3234. 10.1128/AEM.71.6.3228 15933025 PMC1151843

[34] Lieberman TD, Michel J-B, Aingaran M, Potter-Bynoe G, Roux D, Davis MR Jr, et al (2011) Parallel bacterial evolution within multiple patients identifies candidate pathogenicity genes. Nat Genet 43: 1275–1280. Available: http://www.pubmedcentral.nih.gov/articlerender.fcgi?artid=3245322&tool=pmcentrez&rendertype=abstract. Accessed 21 March 2014. 10.1038/ng.997 22081229 PMC3245322

[35] Cooper TF, Rozen DE, Lenski RE (2003) Parallel changes in gene expression after 20,000 generations of evolution in *Escherichia coli*. PNAS 100: 1072–1077. 12538876 10.1073/pnas.0334340100 PMC298728

[36] Woods RJ, Barrick JE, Cooper TF, Shrestha U, Kauth MR, Lenski RE. (2011) Second-order selection for evolvability in a large *Escherichia coli* population. Science 331: 1433–1436. Available: http://www.pubmedcentral.nih.gov/articlerender.fcgi?artid=3176658&tool=pmcentrez&rendertype=abstract. Accessed 21 March 2014. 10.1126/science.1198914 21415350 PMC3176658

[37] Plucain J, Hindré T, Le Gac M, Tenaillon O, Cruveiller S, Médigue C, et al (2014) Epistasis and allele specificity in the emergence of a stable polymorphism in *Escherichia coli*. Science 343: 1366–1369. Available: http://www.ncbi.nlm.nih.gov/pubmed/24603152. Accessed 20 March 2014. 10.1126/science.1248688 24603152

[38] Chubiz LM, Granger BR, Segrè D, and Harcombe WR 2015 Species interactions differ in their genetic robustness. Front Microbiol. 6:271 10.3389/fmicb.2015.00271 25926820 PMC4396422

[39] Momeni B, Brileya KA, Fields MW, Shou W. 2013 Strong inter-population cooperation leads to partner intermixing in microbial communities. eLife 2:e00230 10.7554/eLife.00230 23359860 PMC3552619

[40] Marx CJ (2013) Can you sequence ecology? Metagenomics of adaptive diversification. PLoS Biol 11: e1001487 Available: http://www.pubmedcentral.nih.gov/articlerender.fcgi?artid=3576389&tool=pmcentrez&rendertype=abstract. Accessed 23 May 2014. 10.1371/journal.pbio.1001487 23431268 PMC3576389

[41] Delaney NF, Kaczmarek ME, Ward LM, Swanson PK, Lee M-C, Marx CJ. (2013) Development of an optimized medium, strain and high-throughput culturing methods for *Methylobacterium extorquens*. PLoS One 8: e62957 Available: http://www.pubmedcentral.nih.gov/articlerender.fcgi?artid=3639900&tool=pmcentrez&rendertype=abstract. Accessed 24 April 2014. 10.1371/journal.pone.0062957 23646164 PMC3639900

[42] Barrick JE, Yu DS, Yoon SH, Jeong H, Oh TK, Schneider D, et al (2009) Genome evolution and adaptation in a long-term experiment with *Escherichia coli*. Nature 461: 1243–1247. Available: http://www.ncbi.nlm.nih.gov/pubmed/19838166. Accessed 19 March 2014. 10.1038/nature08480 19838166

[43] Datsenko KA, Wanner BL (2000) One-step inactivation of chromosomal genes in *Escherichia coli* K-12 using PCR products. PNAS 97: 6640–6645. Available: http://www.pubmedcentral.nih.gov/articlerender.fcgi?artid=18686&tool=pmcentrez&rendertype=abstract. 10829079 10.1073/pnas.120163297 PMC18686

[44] Arnold K, Bordoli L, Kopp J, Schwede T (2006) The SWISS-MODEL workspace: a web-based environment for protein structure homology modelling. Bioinformatics 22: 195–201. Available: http://www.ncbi.nlm.nih.gov/pubmed/16301204. Accessed 20 March 2014. 16301204 10.1093/bioinformatics/bti770

[45] Kiefer F, Arnold K, Künzli M, Bordoli L, Schwede T (2009) The SWISS-MODEL Repository and associated resources. Nucleic Acids Res 37: D387–D392. Available: http://www.pubmedcentral.nih.gov/articlerender.fcgi?artid=2686475&tool=pmcentrez&rendertype=abstract. Accessed 25 March 2014. 10.1093/nar/gkn750 18931379 PMC2686475

[46] The PyMOL Molecular Graphics System, Version 1.3 Schrödinger, LLC. (n.d.).

[47] Guerois R, Nielsen JE, Serrano L (2002) Predicting changes in the stability of proteins and protein complexes: a study of more than 1000 mutations. J Mol Biol 320: 369–387. 10.1016/S0022-2836(02)00442-4 12079393

[48] Schymkowitz JWH, Rousseau F, Martins IC, Ferkinghoff-Borg J, Stricher F, Serrano L. (2005) Prediction of water and metal binding sites and their affinities by using the Fold-X force field. Proc Natl Acad Sci U S A 102: 10147–10152. 10.1073/pnas.0501980102 16006526 PMC1177371

[49] Miller CR, Lee KH, Wichman HA, Ytreberg FM (2014) Changing folding and binding stability in a viral coat protein: a comparison between substitutions accessible through mutation and those fixed by natural selection. PLoS One 9: e112988 Available: http://dx.plos.org/10.1371/journal.pone.0112988. 10.1371/journal.pone.0112988 25405628 PMC4236103

[50] Lee KH, Miller CR, Nagel AC, Wichman HA, Joyce P, Ytreberg FM. (2011) First-step mutations for adaptation at elevated temperature increase capsid stability in a virus. PLoS One 6 10.1371/journal.pone.0025640 PMC3183071 21980515

[51] Zhan YA, Wu H, Powell AT, Daughdrill GW, Ytreberg FM (2013) Impact of the K24N mutation on the transactivation domain of p53 and its binding to murine double-minute clone 2. Proteins Struct Funct Bioinforma 81: 1738–1747. 10.1002/prot.24310 PMC4160123 23609977

[52] Shyu C, Cavileer TD, Nagler JJ, Ytreberg FM (2011) Computational estimation of rainbow trout estrogen receptor binding affinities for environmental estrogens. Toxicol Appl Pharmacol 250: 322–326. Available: 10.1016/j.taap.2010.11.005 21075131 PMC3022107

[53] Ellermeier CD, Janakiraman A, Slauch JM (2002) Construction of targeted single copy *lac* fusions using lambda Red and FLP-mediated site-specific recombination in bacteria. Gene 290: 153–161. Available: http://www.ncbi.nlm.nih.gov/pubmed/12062810. 12062810 10.1016/s0378-1119(02)00551-6

[54] Zamboni N, Fendt S-M, Rühl M, Sauer U (2009) (13)C-based metabolic flux analysis. Nat Protoc 4: 878–892. Available: http://www.ncbi.nlm.nih.gov/pubmed/19478804. Accessed 20 March 2014. 10.1038/nprot.2009.58 19478804

[55] Delaney NF, Rojas Echenique JI, Marx CJ (2013) Clarity: an open-source manager for laboratory automation. J Lab Autom 18: 171–177. 10.1177/2211068212460237 23032169 PMC5170844

