# Supplementary material for: Parallel Mutations Result in a Wide Range of Cooperation and Community Consequences in a Two-Species Bacterial Consortium
Source: PLoS One. 2016 Sep 12;11(9):e0161837. doi: 10.1371/journal.pone.0161837 (PMC5019393; doi:10.1371/journal.pone.0161837)
Supplement: S1 Fig — Wild-type with the evolved alleles of metA cooperate more than with the original allele, but significantly less than the respective evolved isolates. Asterisks indicate presence of native allele of that background (i.e., no substitution). (DOCX) [file pone.0161837.s001.docx]

**S1 Figure. Consortia growth dependence upon *metA* alleles of *S. enterica*.** Wild-type with the evolved alleles of *metA* cooperate more than with the original allele, but significantly less than the respective evolved isolates. Asterisks indicate presence of native allele of that background (i.e., no substitution).
