# Supplementary material for: Parallel Mutations Result in a Wide Range of Cooperation and Community Consequences in a Two-Species Bacterial Consortium
Source: PLoS One. 2016 Sep 12;11(9):e0161837. doi: 10.1371/journal.pone.0161837 (PMC5019393; doi:10.1371/journal.pone.0161837)
Supplement: S2 Fig — YFP-labeled S. enterica and CFP-labeled E. coli ΔmetB grown liquid media were sampled at various time points, and consortia composition determined via flow-cytometery. Each data point represents three biological replicates, each with three technical replicates. (DOCX) [file pone.0161837.s002.docx]

**S2 Figure. Consortia composition reaches an equilibrium with time.** YFP-labeled *S. enterica* and CFP-*labeled E. coli* Δ*metB* grown liquid media were sampled at various time points, and consortia composition determined via flow-cytometery. Each data point represents three biological replicates, each with three technical replicates.
