# Supplementary material for: Parallel Mutations Result in a Wide Range of Cooperation and Community Consequences in a Two-Species Bacterial Consortium
Source: PLoS One. 2016 Sep 12;11(9):e0161837. doi: 10.1371/journal.pone.0161837 (PMC5019393; doi:10.1371/journal.pone.0161837)
Supplement: S3 Fig — This sample is one biological replicate of R1P2, with the methionine and isoleucine peaks clearly visible. Fragment patterns at each peak match those of identified derivatized amino acids. Area under each peak corresponds to amino acid quantity. (DOCX) [file pone.0161837.s003.docx]

**S3 Figure. Example GC-chromatogram of derivatized amino acids from cooperator spent media**. This sample is one biological replicate of R1P2, with the methionine and isoleucine peaks clearly visible. Fragment patterns at each peak match those of identified derivatized amino acids. Area under each peak corresponds to amino acid quantity.
