# Supplementary material for: Parallel Mutations Result in a Wide Range of Cooperation and Community Consequences in a Two-Species Bacterial Consortium
Source: PLoS One. 2016 Sep 12;11(9):e0161837. doi: 10.1371/journal.pone.0161837 (PMC5019393; doi:10.1371/journal.pone.0161837)
Supplement: S1 Table — (DOCX) [file pone.0161837.s004.docx]

**S1 Table – Strain and plasmid list**

| **Strain Number** | **Strain Name** | **Description** |
| --- | --- | --- |
| CM4322 | *E. coli* Δ*metB* | *∆(araD-araB) ∆lacZ4787(::rrnB-3) λ- ∆(rhaBAD)568 rph-1 hsdR514 ∆metB726::kan* |
| CM4323 | LT2 | *S. enterica* serovar *Typhimurium* str. LT2 |
| CM4324 | 14028s | *S. enterica* serovar *Typhimurium* str. 14028s |
| CM4325 | R1 | LT2 *metJ*-16::IS10 |
| CM4326 | R2 | 14028s *metJ*^R2^ |
| CM4327 | R3 | 14028s *metJ*^R3^ |
| CM4328 | R1P1 | LT2 *metJ*-16::IS10 *metA*^P1^ |
| CM4329 | R1P2 | LT2 *metJ*-16::IS10 *metA*^P2^ |
| CM4330 | R1P3 | LT2 *metJ*-16::IS10 *metA*^P3^ |
| CM4331 | R2P4 | 14028s *metJ*^R2^ *metA*^P4^ |
| CM4332 | R3P5 | 14028s *metJ*^R3^ *metA*^P5^ |
| CM4333 | R3P6 | 14028s *metJ*^R3^ *metA*^P6^ |
| CM4334 | R3P7 | 14028s *metJ*^R3^ *metA*^P6^ |
| CM4335 | R3P8 | 14028s *metJ*^R3^ *metA*^P6^ |
| CM4336 | LT2 ∆*metA* | LT2 ∆*metA*::*cat* |
| CM4337 | LT2 *metA^P1^* | R1P1 X LT2 ∆*metA*::*cat* |
| CM4338 | LT2 *metA^P4^* | R2P4 X LT2 ∆*metA*::*cat* |
| CM4339 | R1∆*metA* | R1 ∆*metA*::*cat* |
| CM4340 | R2 ∆*metA* | R2 ∆*metA*::*cat* |
| CM4341 | R3 ∆*metA* | R3 ∆*metA*::*cat* |
| CM4342 | R1 *metA^WT^* | R1 X R1∆*metA* |
| CM4343 | R1 *metA^R1P1^* | R1P1 X R1∆*metA* |
| CM4344 | R1 *metA^R1P2^* | R1P2 X R1∆*metA* |
| CM4345 | R1 *metA^R1P3^* | R1P3 X R1∆*metA* |
| CM4346 | R2 *metA^R2P4^* | R2P4 X R2∆*metA* |
| CM4347 | R3 *metA^R3P5^* | R3P5 X R3∆*metA* |
| CM4348 | R3 *metA^R3P6^* | R3P7 X R3∆*metA* |
| CM4349 | R3 *metA^R3P7^* | R3P8 X R3∆*metA* |
| CM4350 | LT2 FLAG | LT2 *metA*'-'[::3xFLAG *kan*](hyb) |
| CM4351 | R1 FLAG | R1 *metA*'-'[::3xFLAG *kan*](hyb) |
| CM4352 | R1+Myc | R1 *metA*'-'Myc(hyb) |
| CM4353 | R2+Myc | R2 metA'-'Myc(hyb) |
| CM4354 | R2+NHA | R1 HA'-'*metA*(hyb) |
| CM4355 | R1+NHA | R2 HA'-'*metA*(hyb) |
| CM4356 | CFP *E. coli* Δ*metB* | *E. coli* Δ*metB* ϕ80 *attB::*CFP |
| CM4357 | YFP R1P1 | R1P1 l *λ attB::pVenus* |
| CM4358 | YFP R1P2 | R1P2 l *λ attB::pVenus* |
| CM4359 | YFP R1P3 | R1P3 l *λ attB::pVenus* |
| CM4360 | YFP R3P6 | R3P6 *λ attB::pVenus* |
| CM4361 | YFP R3P7 | R3P7 *λ* *attB::pVenus* |
| LC1589 | LC1589 | LT2 *λ* *attB*::pLC246 |
| LC1511 | LC1511 | MG1655 *attB* ϕ80::pLC249 |
| plasmid | pKD46 | *P_araBAD'-exo-beta-gam t0 oriSC101(ts) bla* |
| plasmid | pLC246 | *rrnB P_L'-venus t0 oriR6K kan* |
| plasmid | pLC249 | *rrnB P_L'-cerulean t0 oriR6K cat* |
