# Supplementary material for: Parallel Mutations Result in a Wide Range of Cooperation and Community Consequences in a Two-Species Bacterial Consortium
Source: PLoS One. 2016 Sep 12;11(9):e0161837. doi: 10.1371/journal.pone.0161837 (PMC5019393; doi:10.1371/journal.pone.0161837)
Supplement: S2 Table — (DOCX) [file pone.0161837.s005.docx]

**S2 Table – Primers used in gene disruptions, replacements, and epitope tagging.**

| **Primer Name** | **Sequence** | **Template** | **Function** |
| --- | --- | --- | --- |
| ∆metA::cat F | ggatgtctaaacgtttaaacgtatgtcgtgaggttatcag gtgtaggctggagctgcttc | pKD32 (Datsenko and Wanner 2000) | *metA* deletion |
| ∆metA::cat R | aggcacccgaaggTGCCTGATTCAACATGCTGAAACGCTT ctgtcaaacatgagaattaa | pKD32 | *metA* deletion |
| SMD22 | gatcttccgtcacaggtagg | ∆*metA::cat* | internal cat primer to check insertion |
| SMD8 | CTCACCTTGAACTTGCAGAC | chromosomal *metA* | amplify *metA* F |
| SMD9 | ATGCGAACGAAGGATCACTG | chromosomal *metA* | amplify *metA* R |
| SMD1 | TACGCCATATGATCTGCGTCACATGAATCCAACCCTGGATGACTACAAAGACCATGACGG | pSUB11 (Uzzau et al. 2001) | 3xFLAG HTS |
| SMD4 | CAACATGCTGAAACGCTTTAGGATCGTCAGAAGCAGAAGACA TAT GAA TAT CCT CCT TAG | pSUB11 | 3xFLAG HTS |
| SMD40 | TTGGATCCCGAACTGGAAGAACTGGTGAG | chromosomal *metA* | pKNOCK-*metA* cloning |
| SMD41 | TTGGATCCCTCACCAGTTCTTCCAGTTCG | chromosomal *metA* | pKNOCK-*metA* cloning |
| SMD44 | ACGTGTTCCGCTTCCTTTAG | pKNOCK-*metA* | external pKNOCK R to check insertion |
| SMD50 | TTGGTCTCA GAGATCAGTTTCTGTTC ATCCAGGGTTGGATTCATGTGACG | pKNOCK (Alexeyev 1999) | EI-PCR construction of MyC HTS R |
| SMD51 | TTGGTCTCATCTCTGAAGAAGACCTGTAATCTTCTGCTTCTGACGATCCT | pKNOCK | EI-PCR construction of MyC HTS F |
| SMD52 | TTGGTCTCAGGAACATCATACGGATAATCCAGGGTTGGATTCATGTGACG | pKNOCK | EI-PCR construction of HTS HA tag R (C terminus) |
| SMD53 | TTGGTCTCATTCCTGATTATGCTAGCCTCTAATCTTCTGCTTCTGACGATCCT | pKNOCK | EI-PCR construction of HTS HA tag F (C terminus) |
| SMD54 | TTGGTCTCAGGAACATCATACGGATACATAACCTGATAACCTCACGAC | pKNOCK | EI-PCR construction of HTS HA tag R (N terminus) |
| SMD55 | TTGGTCTCA TTCCTGATTATGCTAGCCTC CCGATTCGCGTGCTGGAC | pKNOCK | EI-PCR construction of HTS HA tag F (N terminus) |

**References:**

Alexeyev MF (1999) The pKNOCK series of broad-host-range mobilizable suicide vectors for gene knockout and targeted DNA insertion into the chromosome of Gram-negative bacteria. *Biotechniques* 26:824–827.

Datsenko KA, Wanner BL (2000) One-step inactivation of chromosomal genes in *Escherichia coli* K-12 using PCR products. *PNAS* 97:6640–6645.

Uzzau S, Figueroa-Bossi N, Rubino S, Bossi L (2001) Epitope tagging of chromosomal genes in *Salmonella*. *PNAS* 98:15264–15269.
